# Case report: A *de novo* variant of CRMP1 in an individual with a neurodevelopmental disorder

**DOI:** 10.3389/fnins.2024.1490731

**Published:** 2024-12-20

**Authors:** Juan Liu, Qi Wang, Jia Chen

**Affiliations:** ^1^Department of Pediatrics, Mianyang Central Hospital, Mianyang, Sichuan, China; ^2^Chengdu Medical College, Chengdu, Sichuan, China

**Keywords:** CRMP1, neurodevelopmental disorder, whole-exome sequencing, autism spectrum disorder, children

## Abstract

**Background:**

CRMP1 is a key protein involved in brain development.

**Methods:**

We performed genetic testing through whole-exome sequencing (WES) in an individual with a neurodevelopmental disorder.

**Results:**

We identified a *de novo* heterozygous *CRMP1* NM_001014809.3:c.1755del (p.Lys586fs) variant in the affected individual. This mutation was submitted to ClinVar (SCV005196589).

**Conclusion:**

Currently, the *CRMP1* gene has no clear disease phenotype association in the Online Mendelian Inheritance in Man (OMIM) database. Our report may provide evidence for an association between the *CRMP1* gene and neurodevelopmental disorders (NDDs).

## 1 Introduction

Neurodevelopmental disorders(NDDs) are a group of chronic developmental brain disorders, including autism spectrum disorder(ASD), intellectual developmental disorder (IDD), developmental speech or language disorder (DLD), attention-deficit/hyperactivity disorder (ADHD), and tic disorders (Zhu et al., 2023). The prevalence of NDDs among individuals under the age of 18 years, as defined by the *Diagnostic and Statistical Manual of Mental Disorders*, Fifth Edition (DSM-5) criteria, has been infrequently measured. Existing studies have reported the following prevalence rates: ASD has a prevalence rate ranging from 0.70 to 3% of the population, ID occurs at a rate of 0.63%, ADHD has a prevalence rate ranging from 5 to 11%, among other conditions (Shen et al., 2020). The pathogenesis of NDDs is not fully understood; however, it is mainly caused by genetic and neurobiological factors and shows a trend of continuous development (von Gontard et al., 2022).

Collapsin response mediator proteins (CRMPs), also known as dihydropyrimidinase-like (DPYSL) proteins, members of the cytoplasmic phosphoprotein family, consist of five cytoplasmic phosphoproteins (CRMP1-5) that are widely expressed in the nervous system and are essential for brain development and function. They play crucial roles in cellular processes, such as cell migration, neurite extension, axonal guidance, dendritic spine development, and synaptic plasticity, through their phosphorylation status (Desprez et al., 2023). CRMP2, the first protein of the CRMP family to be cloned, was identified as an intracellular messenger required for Sema3A-induced growth cone collapse (Charrier et al., 2003). It has also been shown that CRMP3-deficient (*CRMP3*^−/−^) mice exhibit abnormal dendritic fluctuations, confirming the importance of CRMP3 in hippocampal dendritic organization (Quach et al., 2008). CRMP1, also known as UNC-33-like phosphoprotein 1 (ULIP1), is involved in signaling protein-induced growth cone collapse during neurodevelopment, as part of the protein signal transduction pathway in the brain. CRMP1 is involved in the dendritic development of cortical pyramidal neurons, and it also mediates the transmission of the reelin signal in cortical neuron migration and regulates the migration of cerebral cortical neurons (Kawashima et al., 2021; Yamashita et al., 2006). Given their key function in developmental processes, disturbances in CRMP function can result in neurodevelopmental diseases (Ravindran et al., 2022).

At present, there are few reports on *CRMP1* and human neuropsychiatric disorders. In chronic brain diseases, such as Alzheimer’s disease and Parkinson’s disease, pathological features include misfolding or abnormal aggregation of various proteins, including CRMP1 (Ochneva et al., 2022). In 2012, Verian Bader et al. demonstrated that lymphoblastic cell lines from patients with schizophrenia showed increased CRMP1 expression (Bader et al., 2012). Researchers have also found that maternal autoantibody reactivity to CRMP1 is associated with the severity of ASD (Braunschweig et al., 2013). Da Silva et al. reported that a partial coding sequence of *CRMP1* overlaps with the Ellis-van Creveld (EVC) gene, identifying *CRMP1* as a potential genetic modifier of the severity of EVC syndrome, with approximately 10% of EVC patients possibly exhibiting neurodevelopmental abnormalities (Da Silva et al., 2024). To date, only one report has linked genetic variations in the *CRMP1* gene to neurodevelopmental disorders in humans. This report described a heterozygous *de novo* mutation in the *CRMP1* gene in three unrelated individuals with muscular hypotonia, intellectual impairment, and/or autism spectrum disorder. This mutation is believed to cause neurodevelopmental disorders that affect protein oligomerization and neurite growth (Ravindran et al., 2022).

We present a case of a boy with a heterozygous *de novo* variant in the *CRMP1* gene, identified through whole-exome sequencing (WES), who exhibits phenotypes of a neurodevelopmental disorder such as autism, language delay, hyperactivity, and learning disabilities.

## 2 Methods

### 2.1 Case presentation

A 9-year-old boy was referred to the pediatric behavioral development clinic due to his poor verbal expression and tendency to play alone. He is the first child born to a healthy, non-related couple after a normal pregnancy. He was delivered via cesarean section at full term, with a birth weight of 3,600 g. He was able to walk independently at 15 months old and began speaking at approximately 24 months old.

During kindergarten, he was able to express some ideas using short sentences and understand some common-sense questions, but he had poor eye contact, was not sociable, and lacked awareness of danger. At 7 years of age, he was socially impaired and inattentive. In the hospital, the individual’s Full Scale Intelligence Quotient (FSIQ), as assessed by the Wechsler Intelligence Scale for Children (WISC), was found to be 69, indicating that his cognitive abilities are below the average range for his age group. The conditions mentioned did not improve after the age of 9 years and were characterized by academic deficiency, reduced attention to surroundings, and a limited range of interests, stereotypic behavior, and hypersensitivity to sound or touch.

He came to our hospital for treatment, and the Autism Diagnostic Observation Schedule (ADOS) was used for evaluation. The scores were as follows: 10 points for Social Affect (SR) and one point for Restricted and Repetitive Behaviors (RRB), with a total score above the autism spectrum threshold of seven points. The Autism Diagnostic Interview-Revised (ADI-R) yielded the following scores: 16 points in the Reciprocal Social Interactions domain, 14 points in the Language/Communication domain, and two points in the Restricted, Repetitive, and Stereotyped Behaviors and Interests domain. These scores suggested abnormal communication and the presence of stereotypic behaviors. In the Diagnostic Receptive and Expressive Assessment of Mandarin–Comprehensive (DREAM-C), the individual scored 76 points in the expressive language domain, which was below the normative level of expressive language for his peers. In addition, the FSIQ, as assessed by the WISC, was found to be less than 40, suggesting mild delay. His 25-hydroxyvitamin D3 level was 16.21 ng/mL, and routine metabolic screening showed abnormal results.

### 2.2 Whole-exome sequencing

DNA was obtained from the peripheral blood of the patient and his parents. The DNA was then submitted for trio (proband and parents) whole-exome sequencing (trioWES) to Chigene (Beijing) Translational Medical Research Center Co. Ltd. Protein-coding exome enrichment was performed using xGen Exome Research Panel v2.0 (IDT, Iowa, United States), which consists of 429,826 individually synthesized and quality-controlled probes. This panel targets 39 Mb of the protein-coding region (19,396 genes) of the human genome and covers 51 Mb of end-to-end tiled probe space. High-throughput sequencing was performed using the MGISEQ-T7 series sequencer, with atleast 99% of the target sequence being sequenced. The sequencing process was conducted by Chigene (Beijing) Translational Medical Research Center Co. Ltd.

## 3 Results

**Figure 1:**
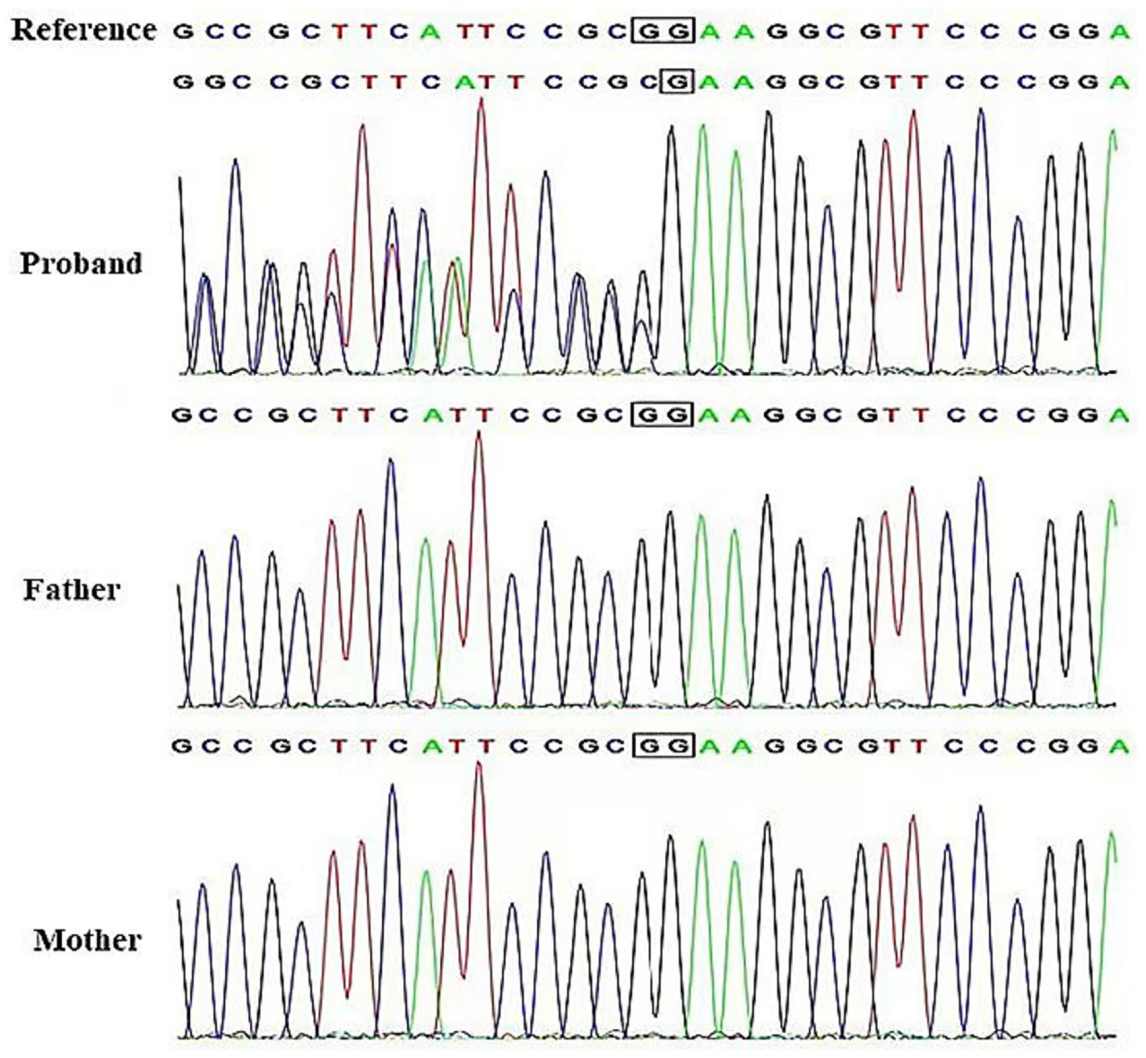
Sanger validation diagram. WT, Wild type.

After obtaining informed consent from the family, genetic testing was conducted. Through WES, a frameshift variant was identified in the proband: NM 001014809.3*(CRMP1)*:c.1755del (p.Lys586fs). The proband is heterozygous for this variant, and both parents are wild-type, indicating that it is a *de novo* mutation. We submitted this mutation to ClinVar (SCV005196589). The variant was confirmed by Sanger sequencing (Figure 1).

**Figure 2:**
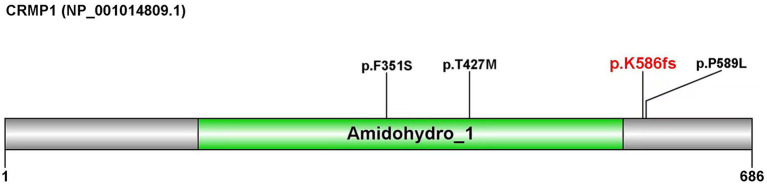
Domain map of the protein encoded by the *CRMP1* gene. The domain was predicted using the Pfam database.

The *CRMP1* (NM_001014809.3) gene contains 14 exons and encodes a protein of 686 amino acids (Figure 2). According to the Pfam database, this protein contains an Amidohydro_1 domain, which belongs to the amidohydrolase family. Amidohydro_1 is a large superfamily of metal-dependent hydrolases (Holm and Sander, 1997). Ravindran et al. (2022) reported three missense variations in the *CRMP1* gene, two of which [NM_001014809.3 (*CRMP1*): c.1280C > T (p.T427M) and NM_001014809.3 (*CRMP1*):c.1052 T > C (p.F351S)] are located within this structure, while the other variation, NM_001014809.3 (*CRMP1*):c.1766C > T (p.P589L), is located downstream of this structure (Ravindran et al., 2022).

## 4 Discussion

The frameshift mutation identified in this report (p.Lys586fs) was located near the p.P589L site.

Neurons are the fundamental structural and functional units of the nervous system, consisting of the soma, membrane, cytoskeleton, dendrites, and axons, and are responsible for information processing and signal transmission. Neural circuit development and adaptability are crucial for nervous system function. CRMPs, highly expressed in the developing nervous system, are integral to neural development, influencing axon guidance, synaptic maturation, cell migration, and adult brain function (Nakamura et al., 2020).

CRMP1 is an intracellular mediator of Sema3A signaling, an important molecule that directs axon outgrowth and branching. By binding to microtubules and F-actin, CRMP1 regulates cytoskeletal depolymerization and reorganization, thereby affecting growth cone movement and orientation (Cai et al., 2017). CRMP1 also plays an important role in synapse formation and maturation. It promotes efficient connectivity between neurons by regulating the organization and function of both presynaptic and postsynaptic components (Nagai et al., 2017). Phosphorylation of CRMP1 by Fyn kinase at Y504 is essential for neuronal migration, regulating growth cone behavior, and axon pathfinding (Buel et al., 2010). As a cyclin-dependent kinase 5 (Cdk5) substrate, CRMP1 is vital for actin cytoskeleton remodeling, which is critical for neuronal development and synaptic plasticity (Shah and Rossie, 2018). The phosphorylation of CRMP1 by kinases such as Cdk5, glycogen synthase kinase-3β (GSK-3β), and Rho-associated kinase (ROCK) alters its conformation, stability, and protein interactions, thereby impacting microtubule dynamics and neuronal morphogenesis (Yamashita and Goshima, 2012). In addition, CRMP1’s localization to the midbody during cytokinesis suggests that it may play a role in the midbody query-query PPI (QQ-PPI) network, influencing the process of abscission and termination of cytokinesis (Chen et al., 2009).

In summary, CRMP1 is a multifaceted protein essential for the development and maintenance of the nervous system. Its roles in axon guidance, dendritic development, neurite outgrowth, and neuronal migration make it a critical factor in the complex orchestration of neurodevelopment. Disruptions in CRMP1’s function, such as those caused by genetic variants, can lead to neurodevelopmental disorders, highlighting the importance of CRMP1 in brain health and function.

We searched the PubMed and Web of Science databases, and found, as mentioned earlier, the only article on the association between CRMP1 and human neurodevelopmental disorders. Through the bioinformatics analysis of three CRMP1 cases and the analysis of the effect of genetic variants on protein structure, it was emphasized that CRMP1 is related to human neurodevelopmental disorders (Ravindran et al., 2022).

**Table 1:** The phenotypic manifestations of the patient in this case and the historical cases.

Characteristics and symptoms	Proband 1	Proband 2	Proband 3	Proband 4
**CRMP1 variant#**	**c.1755delG** p.K586Rfs*75	**c.1766C>T** **p.P589L**	**c.1280C>T;** **p.T427M**	**c.1052T>C;** **p.F351S**
Gender	Male	Female	Male	Female
Weight (kg)	46.3 (1.9 SD)	57.1 (0.15 SD)	N/A	133 (14.2 SD)
Perinatal period	Normal	Normal	Normal	Normal
Macrocephaly (OFC>-2 SD)	Normal	Normal	Normal	+(5 SD)
Delayed motor development	—	**+**	**+**	**+**
Walking unsupported	13 months	28 months	24 months	24 months
Fine motor problems	—	**+**	—	**+**
Delayed speech and language development (first words spoken)	**+** (24 months)	**+** (24 months)	**+** (36 months)	**+** (30 months)
Intellectual disability	Moderate (IQ less than 40)	Moderate (IQ 55)	No (IQ95)	Moderate
Autism spectrum disorder	**+**	—	**+**	—
Behavioral problems	**+**	**+**	**+**	**+**
Auxiliary examinations				
EEG results	Normal	Normal	Normal	Normal
Cranial MRI abnormalities	—	—	—	—

#, NM_001014809.2, NP_0010 14809.1; +, yes; −, no; IQ, intellectual quotient; N/A, not available; SD, standard deviation.

As shown in Table 1, we summarized some common or different characteristics of these four probands. The head MRI of the four cases was normal. Although the cranial MRI findings of the four patients mentioned were within normal limits, recent studies have indicated that MRI remains a beneficial supplementary diagnostic tool in the evaluation of patients with neurodevelopmental disorders (NDDs) of obscure origin. It is especially useful for patients presenting with neurological symptoms or signs, such as motor dysfunction, pyramidal tract disorders, epilepsy, or abnormal head circumference. After conventional metabolic and genetic assessments yield normal results, the diagnostic value of MRI is heightened in these patients as it has the potential to reveal structural brain anomalies that either support or lead to an etiological diagnosis of NDDs (Engbers et al., 2010).

They all had speech and language delays, as well as behavioral problems. Both proband 1 (P1) and proband 3 (P3) were diagnosed with autism. For children with neurodevelopmental disorders, dynamic changes may occur in those with and without ASD. It has been confirmed through a comprehensive behavioral test battery on *CRMP1* knockout (*crmp1*−/−) mice that these mice exhibited hyperactivity, impaired context-dependent memory, and deficits in long-term memory retention (Yamashita et al., 2013). CRMP1 is one of the proteins targeted by maternal autoantibodies in a subset of mothers with children diagnosed with ASD. The presence of these autoantibodies, particularly against CRMP1, has been associated with more severe ASD symptoms, as measured by the ADOS scores (Angkustsiri et al., 2022). SEMA3A is crucial for neurons during the first 13 weeks of pregnancy. CRMP1 is one of the sensors of SEMA3A signaling, and its mutation may affect the normal growth and differentiation of neurons and is also related to ASD (Matrone and Ferretti, 2023).

Interestingly, P2–P4 were reported to have bradykinesia. CRMP1 phosphorylation has been shown to affect motor function in mice. Inhibition of CRMP1 phosphorylation at Ser522, achieved through the use of *Crmp1*^ki/ki^ mice (where the Ser522 phosphorylation site was abolished), led to improved motor function and prolonged survival in SOD1^G93A^ mice. Deletion of both copies of the *Crmp1* gene (*Crmp1*^−/−^) in SOD1^G93A^ mice resulted in deterioration of motor function (Asano et al., 2022). However, our case showed normal motor development, and further studies may be needed to identify the factors contributing to phenotypic heterogeneity.

The P1 we report exhibited a broader range of phenotypes, and he was treated as follows:

1. Medicinal Applications: Extended-release methylphenidate hydrochloride tablets, phosphatidylserine, and probiotic active freeze-dried powder.
2. Rehabilitation Training: Integrating functional magnetic resonance imaging with computer analysis and localization for precise targeted transcranial magnetic stimulation therapy, auditory integration training, Applied Behavior Analysis (ABA) training guidance, and comprehensive rehabilitation training at rehabilitation institutions.
3. Home Rehabilitation Therapy: Fostering self-esteem and self-confidence, cultivating attention and a sense of achievement, ensuring reasonable allocation of family time, assisting in in the development of good study and living habits, promoting the development of life skills, and encouraging learning and participation in household chores.

Following the systematic treatment, the child demonstrated increased eye contact compared to before, improved completion of two- or three-steps, increased initiation of communication when needed, richer content in questions asked, more complete sentence structure compared to before, the ability to perceive the emotions of family members, attention to the movements of people around, and the capacity to make simple responses. There was also an improvement in puzzle-solving and computational skills, along with a slight improvement in attention.

## 5 Conclusion

Currently, the *CRMP1* gene has no clear disease phenotype association in the OMIM database. The *CRMP1* (p.K586Rfs*75) frameshift variant was identified in this patient, with the following phenotypes: autism/autism spectrum disorder, stereotypic behavior, social impairment, delayed speech and language development, poor eye contact, and attention deficit hyperactivity disorder. Our report may provide evidence for an association between the *CRMP1* gene and neurodevelopmental disorders.

## Data Availability

The data presented in the study are deposited in the ClinVar repository, accession number SCV005196589. The link to the repository is https://submit.ncbi.nlm.nih.gov/clinvar/.
